# Multi-centre evaluation of real-time multiplex PCR for detection of carbapenemase genes OXA-48, VIM, IMP, NDM and KPC

**DOI:** 10.1186/1471-2334-14-27

**Published:** 2014-01-14

**Authors:** Anneke van der Zee, Lieuwe Roorda, Gerda Bosman, Ad C Fluit, Mirjam Hermans, Paul HM Smits, Adri GM van der Zanden, René te Witt, Lesla ES Bruijnesteijn van Coppenraet, James Cohen Stuart, Jacobus M Ossewaarde

**Affiliations:** 1 Maasstad Laboratory, Molecular Diagnostics Unit, Maasstad Hospital, Rotterdam, The Netherlands; 2 Department of Medical Microbiology, University Medical Center Utrecht, Utrecht, The Netherlands; 3 Molecular Diagnostics, Jeroen Bosch Hospital, ‘s-Hertogenbosch, The Netherlands; 4 Molecular Biology Laboratory, Slotervaart Hospital, Amsterdam, The Netherlands; 5 Laboratory for Microbiology and Public Health, Enschede, The Netherlands; 6 Department of Medical Microbiology and Infectious Diseases, Erasmus MC, Rotterdam, The Netherlands; 7 Isala clinics, Laboratory for Medical Microbiology and Infectious Diseases, Zwolle, The Netherlands; 8 Medical Centre Alkmaar, Alkmaar, The Netherlands

**Keywords:** Real-time multiplex PCR, Carbapenemases, OXA-48, VIM, IMP, NDM, KPC

## Abstract

**Background:**

Resistance to carbapenem antibiotics is emerging worldwide among Enterobacteriaceae. To prevent hospital transmission due to unnoticed carriage of carbapenemase producing micro-organisms in newly admitted patients, or follow-up of patients in an outbreak setting, a molecular screening method was developed for detection of the most prevalent carbapenemase genes; bla_OXA-48_, bla_VIM_, bla_IMP_, bla_NDM_ and bla_KPC_.

**Methods:**

A real-time multiplex PCR assay was evaluated using a collection of 86 Gram negative isolates, including 62 carbapenemase producers. Seven different laboratories carried out this method and used the assay for detection of the carbapenemase genes on a selection of 20 isolates.

**Results:**

Both sensitivity and specificity of the multiplex PCR assay was 100%, as established by results on the strain collection and the inter-laboratory comparisons.

**Conclusions:**

In this study, we present a multiplex real-time PCR that is a robust, reliable and rapid method for the detection of the most prevalent carbapenemases bla_OXA-48_, bla_VIM_, bla_IMP_, bla_NDM_ and bla_KPC_, and is suitable for screening of broth cultured rectal swabs and for identification of carbapenemase genes in cultures.

## Background

Carbapenemase producing Enterobacteriaceae (CPE) are emerging worldwide, and have been implicated in numerous outbreaks [1-3]. Rapid and accurate detection of CPE is pivotal for adequate antibiotic therapy and infection control, especially in an outbreak setting. The most commonly used phenotypic CPE confirmation tests, the modified Hodge test and the carbapenemase inhibition tests with boronic acid or EDTA/DPA, have several disadvantages, because those tests require an overnight incubation step, do not provide information on the carbapenemase gene, and cannot differentiate OXA-48 producing isolates from ESBL and/or AmpC producing isolates with decreased permeability [4-7]. Finally, phenotypic detection of CPE may be difficult because carbapenem MICs may be low (in the susceptible range), especially of OXA-48 producing Enterobacteriaceae. Therefore, genotypic detection of carbapenemase genes is the gold standard, although it only detects a pre-specified set of known genes. Here, we describe a real-time PCR for detection of NDM, KPC, VIM, IMP and OXA-48 genes, which are currently the most prevalent carbapenemases [8]. The main goal of this study was optimization of this real time PCR and to determine the test characteristics on a set of well characterized isolates. An interlaboratory performance comparison of the PCR assay was initiated to investigate its robustness and reliability.

## Methods

### PCR design

We developed specific real-time PCRs for detection of KPC, VIM, NDM, OXA-48 and IMP. For design of the primers, sequence variations of carbapenemase genes published at http://www.lahey.org/Studies were taken into account, along with synonymous mutations (Additional file 1: Table S1). Detection of CTX-M [9] was included for optional use since it complements OXA-48 for resistance to extended spectrum cephalosporins, a characteristic that is intrinsic to the other carbapenemases. For verification of newly designed PCRs, sequencing was done on larger gene fragments. For NDM sequencing primers 5′-GCGAAAGTCAGGCTGTGTTG-3, and ‘5′-CATTAGCCGCTGCATTGATG-3′, were used, and for IMP sequencing primers 5′-GGCGGAATAGAGTGGCTTAATTCTC-3′, and 5′-CGTACGGTTTAACAAAACAACCACC-3′ For each separate carbapenemase gene, PCR primers and probe concentrations were optimized. Multiplex combinations were compared with single PCRs in presence of the internal control Phocine Herpes Virus (PhHV).

### Bacterial strains

PCRs were optimized and validated using the following control strains: a KPC producing *Klebsiella pneumonia*, a VIM-2 producing *Pseudomonas aeruginosa*, an IMP-18 producing *P. aeruginosa*, an IMP-28 producing *K. pneumoniae*, a NDM-1 positive *K. pneumoniae*, and an OXA-48 positive *K. pneumoniae* isolate.

Testing of PCR specificity was carried out on the following strain collection. The test collection of 86 isolates, included 58 carbapenemase producing isolates*,* and 28 carbapenemase negative controls. The 58 carbapenemase positive isolates consisted of 45 *K. pneumoniae,* 4 *E. coli,* 3 *Enterobacter* species*, 2 P. mirabilis,* 2 *Citrobacter* species, and 2 *P. aeruginoasa* isolates producing the following carbapenemases: 20 KPC-2/3, 4 KPC plus VIM, 21 VIM, 4 NDM-1, 2 IMP, and 7 OXA-48. The 28 carbapenemase negative controls consisted of 10 *K. pneumoniae*, 2 *E. coli* and 16 *Enterobacter* isolates, producing either an ESBL (20 isolates) or an AmpC beta-lactamase (Additional file 2: Table S2). As the reference test for presence of beta-lactamases, PCR and sequencing was used [10].

### PCR evaluation

To determine the sensitivity and specificity of PCR, 86 unrelated test isolates were investigated both from agar plate and broth. Two protocols were followed; protocol 1) simultaneous multiplex detection of OXA-48, VIM, IMP, NDM and KPC. In this PCR reaction, the OXA-48 probe was labelled with FAM, and the other carbapenemases with VIC. Protocol 2) 3 multiplex PCRs for identification of respectively OXA-48/CTX-M, VIM/IMP, and NDM/KPC. PhHV was labelled with NED. Fluorescent labels of carbapenemase genes were FAM, and VIC, respectively (Additional file 3: Table S3).

### PCR

Strains were grown on MacConkey agar (Oxoid), and in Brain Heart broth, both supplemented with ertapenem (0.125 mg/l). One colony was taken from the plate and 50 μl from the broth. Both were suspended in 100 μl Extraction Solution (SIGMA, E7526). Mixtures were incubated at 95°C for 10 minutes, cooled to room temperature, 100 μl Dilution Buffer (SIGMA, D5688) was added and mixed. PCR reactions were carried out using PCR-ReadyMix™ (SIGMA, E3004). Amplification was performed on ABI 7500 Real-Time PCR system (LifeTech, Glasgow, UK). The temperature profile included initial denaturation of 4 min. at 94°C, followed by 50 cycles (40 cycles, protocol 2) of 94°C for 15 sec., and 60°C for 1 min.

## Results

### Evaluation of PCR

**Figure 1:**
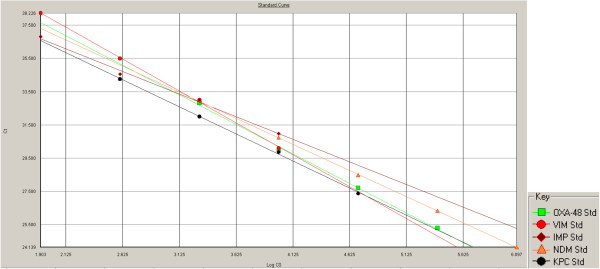
**Standard curve of multiplex PCR.** Inferred efficiencies of multiplex amplification range from 82% for IMP, to 85% for VIM.

PCRs on serial dilutions of template showed Cq (Cycle threshold) values ranging from 19.5-41.5 for single PCRs, and Cq values of 20.2-46.3 for multiplex reactions. The mean difference in Cq value between single and multiplex reactions was 0.9, with a tendency towards an increased difference with decreasing template concentrations. Mixing of templates did not affect Cq values. The lower limit of detection was approximately 10 colony forming units. The amplification efficiencies in multiplex format ranged from 85% for VIM to 82% for IMP (Figure 1). We concluded that the PCRs were compatible.

**Figure 2:**
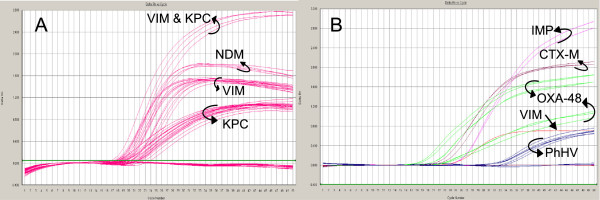
**A) Amplification plot of VIC labelled PCR products of VIM, NDM, and KPC (protocol 1), and B) OXA-48/CTX-M and VIM/IMP (protocol 2).** Reactions were performed in the presence of the internal control PhHV. For clarity, a selection of strains is shown. OXA-48 positives with lower plots were also positive for CTX-M.

The PCR assay according to protocol 1 was carried out on the test collection of 86 strains (Additional file 2: Table S2). All carbapenemase producing isolates were positive in the PCR with Cq values ranging from 15.4 to 23 for isolates from agar, and Ct values 20.1 to 28 from broth cultures (Figure 2A), corresponding to a sensitivity of 100%. All 26 carbapenemase negative control isolates were negative in the PCR, corresponding to a specificity of 100%.

To evaluate the capacity of the real-time PCR to identify carbapenemase genes, protocol 2 was used. Twenty-five strains were PCR positive for VIM, 2 for IMP, 4 for NDM, 24 for KPC, 7 for OXA-48 and 18 for CTX-M. Four strains were positive for both VIM and KPC and 2 strains both for OXA-48 and CTX-M (Figure 2B). Ct values ranged from 15.1-22.5 (VIM), 13.2-20.6 (KPC), 17.8-20.1 (NDM), 16.8-23.8 (OXA-48) and, 14.7-17.2 (CTX-M). The results were 100% concordant with listed strain characteristics.

### Multi-centre comparison

The PCR assay (protocol 2) was tested on a selection of 20 strains (Table 1) by 7 different laboratories. Different PCR platforms were used; ABI7500 (Life Technologies), RotorgeneQ (Qiagen), Biorad CFX96 (Bio-Rad), and Lightcycler 480 (Roche). Also different DNA polymerases were used; PCR ReadyMix (Sigma), Platinum® Multiplex PCR Master Mix (Life Technologies), Fast start master (Roche), and Master Mix for PCR (Bio-Rad). All laboratories correctly detected the targeted carbapenemase genes, and no false positive results were found. With exception of CTX-M, which was not tested, or partially tested as is shown in Table 1, results of the multi-centre comparison were all concordant and again, the results were 100% concordant with listed strain characteristics.

**Table 1:** Multi-centre comparison of the PCR assay for detection of carbapenemases, and the PCR platforms, and DNA polymerases used

**Strain ID**	**Laboratory1**	**Laboratory2**	**Laboratory3**^**1**^	**Laboratory4**^**1**^	**Laboratory5**^**2**^	**Laboratory6**^**2**^	**Laboratory7**^**2**^
OXA 8-90	OXA-48	OXA-48	OXA-48	OXA-48	OXA-48	OXA-48	OXA-48
OXA 8-17	OXA-48/CTX-M	OXA-48/CTX-M	OXA-48/CTX-M	OXA-48/CTX-M	OXA-48	OXA-48	OXA-48
S 4-2	OXA-48/CTX-M	OXA-48/CTX-M	OXA-48/CTX-M	OXA-48/CTX-M	OXA-48	OXA-48	OXA-48
S3-60	OXA-48/CTX-M	OXA-48/CTX-M	OXA-48/CTX-M	OXA-48/CTX-M	OXA-48	OXA-48	OXA-48
GR-21/PM-302	VIM	VIM	VIM	VIM	VIM	VIM	VIM
GR-04/KP-69	VIM/KPC	VIM/KPC	VIM/KPC	VIM/KPC	VIM/KPC	VIM/KPC	VIM/KPC
GR-38/KP-139	VIM	VIM	VIM	VIM	VIM	VIM	VIM
GR-31/KP-956	VIM/KPC	VIM/KPC	VIM/KPC	VIM/KPC	VIM/KPC	VIM/KPC	VIM/KPC
GR-23/KP-385	KPC	KPC	KPC	KPC	KPC	KPC	KPC/imp*
New York-11	KPC	KPC	KPC	KPC	KPC	KPC	KPC
New York-3	KPC	KPC	KPC	KPC	KPC	KPC	KPC
JS022	NDM/CTX-M	NDM/CTX-M	NDM/CTX-M	NDM/CTX-M	NDM	NDM	NDM
RC-89	NDM/CTX-M	NDM/CTX-M	NDM/CTX-M	NDM/CTX-M	NDM	NDM	NDM
S 3-62	IMP	IMP	IMP	IMP	IMP	IMP	IMP
S5-36	IMP	IMP	IMP	IMP	IMP	IMP	IMP
RC-79	CTX-M	CTX-M	CTX-M	CTX-M	negative	negative	negative
EIE-UMC-1	CTX-M	CTX-M	negative	negative	negative	negative	negative
RC-8	negative	negative	negative	negative	negative	negative	negative
RC-16	negative	negative	negative	negative	negative	negative	negative
GR-07/KP-3878	negative	negative	negative	negative	negative	negative	negative
thermocycler	Biorad CFX	LC 480	ABI 7500	ABI 7500	LC 480	ABI 7500	LC 480
			Biorad CFX				
polymerase	Sigma	Roche	ABI	ABI	Roche	N-Amp	Roche
			Biorad				

^1^ Only CTX-M group I PCR was performed.

^2^ No CTX-M PCR was performed.

^*^: Background IMP was detected, and scored as inconclusive by laboratory 7.

Thus, both inter- and intra-laboratory, PCR sensitivity and specificity corresponds to 100%. Two laboratories also subjected the 20 control strains to Check-points PCR assay (laboratory 3) and Check-MDR Carba Assay (laboratory 6). The results were concordant with our PCR assay, except that In the Check-MDR Carba Assay IMP-28 was missed.

## Discussion

This assay has several advantages. First, it is able to detect the five most prevalent carbapenemases, whereas previously published real-time PCRs for detection of carbapenemases were designed to detect either exclusively KPC, or exclusively NDM, or a combination of GES, IMI/NMC, KPC, OXA-48 and SME [11-14]. Second, the good performance of the assay when using pre-cultured broth, makes this method suitable for detection of carbapenemases in clinical swabs.

The PCR assays described here were designed to predominantly detect OXA-48 in the follow-up of an outbreak. The PCR assay according to protocol 1 therefore can only detect if one of the other carbapenemases (except OXA-48) is present or not. The probes of the other carbapenemases can also be differentially labelled, making it possible to simultaneously detect and identify the genes. This is however limited to 3–4 different labels in addition to that of the internal control, and depends on the spectral specifics of the type of thermocycler.

Multi-centre evaluation of the assay showed concordant results, which demonstrates that the test is robust and can be performed in different laboratories using different amplification platforms and/or DNA polymerases.

The PCR detection of the IMP gene still requires attention. Check-Points also has withdrawn detection of IMP from their latest kit. Although we designed two probes that in theory can detect all IMP variants, we have not been able to test whether this is indeed the case. This will be subject for future study.

An intrinsic limitation of this type of assay is that new carbapenemase families or new variants of known families may not be detected. The flexibility of the system presented here, however, allows easy adaptation. For example, detection of other genes e.g. PER or GES, might be added to this multiplex PCR.

## Conclusions

The multiplex real-time PCR described here is a robust, reliable and rapid method for detection of the most prevalent carbapenemase genes bla_OXA-48_, bla_VIM_, bla_IMP_, bla_NDM_ and bla_KPC_.

## Competing interests

The authors declare that they have no competing interests.

## Authors’ contributions

AZ conceived of the study, participated in the design, and drafted the manuscript. LR carried out the molecular genetic studies. GB and JMO participated in the design and coordination. The remaining authors coordinated testing and helped to draft the manuscript. All authors read and approved the final manuscript.

## Pre-publication history

The pre-publication history for this paper can be accessed here:

http://www.biomedcentral.com/1471-2334/14/27/prepub

## Supplementary Material

Additional file 1: Table S1 DNA sequences of primers and probes directed against five carbapenemase genes, CTX-M group I-V, and the internal control Phocine Herpes Virus (PhHV) [15]. Click here for file

Additional file 2: Table S2 Investigated isolates and their characteristics. Click here for file

Additional file 3: Table S3 PCR protocol; components and concentration of primers and probes for separate PCRs for screening and determination. Click here for file
