# Supplementary material for: Multi-centre evaluation of real-time multiplex PCR for detection of carbapenemase genes OXA-48, VIM, IMP, NDM and KPC
Source: BMC Infect Dis. 2014 Jan 14;14:27. doi: 10.1186/1471-2334-14-27 (PMC3897903; doi:10.1186/1471-2334-14-27)
Supplement: Additional file 1: Table S1 — DNA sequences of primers and probes directed against five carbapenemase genes, CTX-M group I-V, and the internal control Phocine Herpes Virus (PhHV) [15]. [file 1471-2334-14-27-S1.doc]

**Supplementary**

Table S1. DNA sequences of primers and probes directed against five carbapenemase genes, CTX-M group I-V, and the internal control Phocine Herpes Virus (PhHV)

| template | primer/probe | sequence (5'-3') | reference |
| --- | --- | --- | --- |
| OXA-48 | Forward primer | GCGTGGTTAAGGATGAACAC | according to reference 14 |
|  | Reverse primer | CATCAAGTTCAACCCAACCG | according to reference 14 |
|  | Probe | AGCCATGCTGACCGAAGCCAATG | this study |
| VIM | Forward primer | GAGATTCCCACGCA[C/T]TCTCTAGA | this study |
|  | Reverse primer | AATGCGCAGCACCAGGATAG | according to reference 15 |
|  | Probe | ACGCAGTGCGCTTCGGTCCAGT | this study |
| IMP | Forward primer | GGCGGAATAGAGTGGCTTAATTCTC | modified from reference 14 |
|  | Reverse primer1 | GAATTTTTAGCTTGTACTTTACCGTCTTT | this study |
|  | Reverse primer2 | ATTTTTAGCTTGTACCTTACCGTATT | this study |
|  | Reverse primer3 | TTTGTAGCTTGCACCTTATTGTCTTT | this study |
|  | Probe1 | ATGCATCTGAATTAAC-MGB | this study |
|  | Probe2 | TAT*GCATCT*GAAT*TAA*CAaAT*GA | this study |
| NDM | Forward primer | CATTAGCCGCTGCATTGATG | this study |
|  | Reverse primer | GTCGCCAGTTTCCATTTGCT | this study |
|  | Probe | CATGCCCGGTGAAATCCGCC | this study |
| KPC | Forward primer | TGCAGAGCCCAGTGTCAGTTT | this study |
|  | Reverse primer | CGCTCTATCGGCGATACCA | this study |
|  | Probe | TTCCGTCACGGCGCGCG | modified from reference 12 |
| CTX-M I | Forward primer | GCTGGACTGCCTGCTTCCT |  |
|  | Reverse primer | CGTTGGTGGTGCCATAG[C/T]CA |  |
| CTX-M II | Forward primer | TGCCGAAATCATGGGTAGTG |  |
|  | Reverse primer | TCGTTGGTGGTGCCATAATCT |  |
| CTX-M III | Forward primer | CTACCCACATCGTGGGTTGTC |  |
|  | Reverse primer | GATGTCATTCGTCGTACCATAATCA |  |
| CTX-M IV | Forward primer | ATTCGGGCCGGCTTACC |  |
|  | Reverse primer | ATCATTGGTGGTGCCGTAG[T/C]C |  |
| CTX-M V | Forward primer | ATTCGGGCCGGCTTACC |  |
|  | Reverse primer | GCGATATCATTCGTCGTACCATAA |  |
|  | Probe | CCGCTGCCGGTCTTATC-MGB | according to reference 9 |
| PhHV | Forward primer | GGGCGAATCACAGATTGAATC |  |
|  | Reverse primer | GCGGTTCCAAACGTACCAA |  |
|  | Probe | TTTTTATGTGTCCGCCACCA-MGB |  |

*: LNA (locked nucleic acid)
