# Supplementary material for: Multi-centre evaluation of real-time multiplex PCR for detection of carbapenemase genes OXA-48, VIM, IMP, NDM and KPC
Source: BMC Infect Dis. 2014 Jan 14;14:27. doi: 10.1186/1471-2334-14-27 (PMC3897903; doi:10.1186/1471-2334-14-27)
Supplement: Additional file 2: Table S2 — Investigated isolates and their characteristics. [file 1471-2334-14-27-S2.doc]

Table S2. Investigated isolates and their characteristics

|  | **Strain ID** | **Origin** | **Beta-lactamase** | **Class** | **Species** |
| --- | --- | --- | --- | --- | --- |
| 1 | JS026 | Dutch National BLref lab | KPC-2 | Carbapenemase | K. pneumoniae |
| 2 | JS036 | Dutch National BLref lab | KPC-2 | Carbapenemase | K. pneumoniae |
| 3 | ATCC- BAA-1705 | reference strain | KPC-2 | Carbapenemase | K. pneumoniae |
| 4 | NCTC- 13438 (“438”) | reference strain | KPC-3 | Carbapenemase | K. pneumoniae |
| 5 | RC-0010 | Dutch National BLref lab | KPC-2 & SHV-12 | Carbapenemase | K. pneumoniae |
| 6 | New York-3 | USA, NY | KPC-3 | Carbapenemase | K. pneumoniae |
| 7 | New York-7 | USA, NY | KPC-3 | Carbapenemase | K. pneumoniae |
| 8 | New York-9 | USA, NY | KPC-3 | Carbapenemase | K. pneumoniae |
| 9 | New York-11 | USA, NY | KPC-2 | Carbapenemase | K. pneumoniae |
| 10 | New York-12 | USA, NY | KPC-2 | Carbapenemase | K. pneumoniae |
| 11 | New York-15 | USA, NY | KPC-2 | Carbapenemase | K. pneumoniae |
| 12 | New York-18 | USA, NY | KPC-2 | Carbapenemase | K. pneumoniae |
| 13 | New York-23 | USA, NY | KPC-2 | Carbapenemase | K. pneumoniae |
| 14 | New York-25 | USA, NY | KPC-2 | Carbapenemase | K. pneumoniae |
| 15 | New York-26 | USA, NY | KPC-2 | Carbapenemase | K. pneumoniae |
| 16 | New York-31 | USA, NY | KPC-2 | Carbapenemase | K. pneumoniae |
| 17 | GR-23/KP-385 | Miriagou, Greece | KPC-2 & SHV-5 | Carbapenemase | K. pneumoniae |
| 18 | GR-25/KP-518 | Miriagou, Greece | KPC-2 & SHV-5 | Carbapenemase | K. pneumoniae |
| 19 | GR-27/KP-549 | Miriagou, Greece | KPC-2 & SHV-5 | Carbapenemase | K. pneumoniae |
| 20 | GR-33/KP-971 | Miriagou, Greece | KPC-2 & SHV-5 | Carbapenemase | K. pneumoniae |
| 21 | JS022 | Dutch National BLref lab | NDM-1, CTX-M | Carbapenemase | K. pneumoniae |
| 22 | JS037 | Dutch National BLref lab | NDM-1, CTX-M | Carbapenemase | K. pneumoniae |
| 23 | NCTC- 13443 (“443”) | reference strain | NDM-1, CTX-M | Carbapenemase | K. pneumoniae |
| 24 | NCTC- 13439 | reference strain | VIM-1 | Carbapenemase | K. pneumoniae |
| 25 | GR-19/EC-116 | Miriagou, Greece | CMY-13 & VIM-1 | Carbapenemase | E. coli |
| 26 | GR-20/EC-541 | Miriagou, Greece | CMY-13 & VIM-1 | Carbapenemase | E. coli |
| 27 | GR-21/PM-302 | Miriagou, Greece | CMY-16 & VIM-1 | Carbapenemase | P. mirabilis |
| 28 | GR-22/PM-383 | Miriagou, Greece | CMY-16 & VIM-1 | Carbapenemase | P. mirabilis |
| 29 | Gr-13/KP-700 | Miriagou, Greece | VIM-1 & SHV-5 | Carbapenemase | K. pneumoniae |
| 30 | GR-14/KP-848 | Miriagou, Greece | VIM-1 & SHV-5 | Carbapenemase | K. pneumoniae |
| 31 | GR-15/KP-SEC2 | Miriagou, Greece | VIM-1 | Carbapenemase | K. pneumoniae |
| 32 | GR-16/KP-SEC4 | Miriagou, Greece | VIM-1 | Carbapenemase | K. pneumoniae |
| 33 | GR-17/KP-6/100 | Miriagou, Greece | VIM-1 | Carbapenemase | K. pneumoniae |
| 34 | GR-44/KP-50 | Miriagou, Greece | VIM-1 | Carbapenemase | K. pneumoniae |
| 35 | GR-53/Eclo-SEC5 | Miriagou, Greece | VIM-1 | Carbapenemase | Enterobacter |
| 36 | GR-54/Eclo-SEC6 | Miriagou, Greece | VIM-1 | Carbapenemase | Enterobacter |
| 37 | GR24/KP-505 | Miriagou, Greece | VIM-1 & SHV-5 | Carbapenemase | K. pneumoniae |
| 38 | GR-32/KP-932 | Miriagou, Greece | VIM-1 & SHV-5 | Carbapenemase | K. pneumoniae |
| 39 | GR-38/KP-139 | Miriagou, Greece | VIM-1 & SHV-5 | Carbapenemase | K. pneumoniae |
| 40 | RC-21 | Dutch National BLref lab | VIM-1 | Carbapenemase | K. pneumoniae |
| 41 | RC-22 | Dutch National BLref lab | VIM-1 | Carbapenemase | Enterobacter |
| 42 | RC-48 | Dutch National BLref lab | VIM-1 | Carbapenemase | K. pneumoniae |
| 43 | RC-51 | Dutch National BLref lab | VIM-1 | Carbapenemase | K. pneumoniae |
| 44 | RC-89 | Dutch National BLref lab | NDM-1, CTX-M | Carbapenemase | E. coli |
| 45 | GR-04/KP-69 | Miriagou, Greece | KPC-2&VIM-1&SHV-5 | Carbapenemase | K. pneumoniae |
| 46 | GR-31/KP-956 | Miriagou, Greece | KPC-2&VIM-1&SHV-5 | Carbapenemase | K. pneumoniae |
| 47 | GR-39/KP-240 | Miriagou, Greece | KPC-2&VIM-1&SHV-5 | Carbapenemase | K. pneumoniae |
| 48 | GR-41/KP-383 | Miriagou, Greece | KPC-2&VIM-1&SHV-5 | Carbapenemase | K. pneumoniae |
| 49 | EIE-UMC-1 | Enterobacter BC study | CTX-M-9 | ESBL | Enterobacter |
| 50 | EIE-HAA-2 | Enterobacter BC study | CTX-M-9 | ESBL | Enterobacter |
| 51 | Checkpoints061 | microarray ESBL study | SHV-2 + GES | ESBL | Enterobacter |
| 52 | GR-07/KP-3878 | Miriagou, Greece | GES-6 & SHV-5 | ESBL | K. pneumoniae |
| 53 | NY-EC03 | USA, NY | CMY-2 & ? | AmpC | E. coli |
| 54 | RC-3 | Dutch National BLref lab | CTX-M-15/28 | ESBL | K. pneumoniae |
| 55 | RC-5 | Dutch National BLref lab | SHV-2 | ESBL | K. pneumoniae |
| 56 | RC-8 | Dutch National BLref lab | Derepressed AmpC | AmpC | Enterobacter |
| 57 | RC-16 | Dutch National BLref lab | Derepressed AmpC | AmpC | Enterobacter |
| 58 | RC-18 | Dutch National BLref lab | CTX-M-15 | ESBL | K. pneumoniae |
| 59 | RC-19 | Dutch National BLref lab | CTX-M-15 | ESBL | K. pneumoniae |
| 60 | RC-34 | Dutch National BLref lab | CTX-M-15/28 | ESBL | K. pneumoniae |
| 61 | RC-38 | Dutch National BLref lab | CTX-M-15/28 | ESBL | Enterobacter |
| 62 | RC-41 | Dutch National BLref lab | Derepressed AmpC | AmpC | Enterobacter |
| 63 | RC-42 | Dutch National BLref lab | Derepressed AmpC | AmpC | Enterobacter |
| 64 | RC-55 | Dutch National BLref lab | SHV-12 | ESBL | Enterobacter |
| 65 | RC-58 | Dutch National BLref lab | CTX-M-15 | ESBL | Enterobacter |
| 66 | RC-68 | Dutch National BLref lab | CTX-M-9 & SHV-12 | ESBL | Enterobacter |
| 67 | RC-71 | Dutch National BLref lab | Derepressed AmpC | AmpC | Enterobacter |
| 68 | RC-72 | Dutch National BLref lab | CTX-M-15/28 | ESBL | K. pneumoniae |
| 69 | RC-73 | Dutch National BLref lab | SHV-12 | ESBL | K. pneumoniae |
| 70 | RC-74 | Dutch National BLref lab | Derepressed AmpC | AmpC | Enterobacter |
| 71 | RC-78 | Dutch National BLref lab | CTX-M-9 & SHV-12 | ESBL | Enterobacter |
| 72 | RC-79 | Dutch National BLref lab | CTX-M-15/28 | ESBL | E. coli |
| 73 | RC-82 | Dutch National BLref lab | Derepressed AmpC | AmpC | Enterobacter |
| 74 | RC-88 | Dutch National BLref lab | SHV-12 | ESBL | Enterobacter |
| 75 | S3-60 | Maasstad Hospital | OXA-48, ESBL | Carbapenemase | K. pneumoniae |
| 76 | S3-61 | Maasstad Hospital | OXA-48 | Carbapenemase | K. pneumoniae |
| 77 | S4-90 | Maasstad Hospital | VIM | Carbapenemase | P. aeruginosa |
| 78 | S5-36 | Isala Clinics | IMP-28 | Carbapenemase | K. pneumoniae |
| 79 | OXA 8-90 | Maasstad Hospital | OXA-48 | Carbapenemase | E. coli |
| 80 | OXA 8-24 | Maasstad Hospital | OXA-48 | Carbapenemase | Citrobacter |
| 81 | OXA 8-21 | Maasstad Hospital | OXA-48 | Carbapenemase | Citrobacter |
| 82 | OXA 8-17 | Maasstad Hospital | OXA-48 & CTX-M-15 | ESBL | K. pneumoniae |
| 83 | S 4-2 | P.Nordmann, Paris | OXA-48 & CTX-M-15 | ESBL | K. pneumoniae |
| 84 | S 3-62 | Maasstad Hospital | IMP-18 | Carbapenemase | P. aeruginosa |
| 85 | OXA 3-56 | Maasstad Hospital | OXA-48 | Carbapenemase | K. pneumoniae |
| 86 | OXA 9-95 | Maasstad Hospital | OXA-48 | Carbapenemase | K. pneumoniae |

Strains used for multi-centre evaluation are shaded
