# Supplementary material for: Multi-centre evaluation of real-time multiplex PCR for detection of carbapenemase genes OXA-48, VIM, IMP, NDM and KPC
Source: BMC Infect Dis. 2014 Jan 14;14:27. doi: 10.1186/1471-2334-14-27 (PMC3897903; doi:10.1186/1471-2334-14-27)
Supplement: Additional file 3: Table S3 — PCR protocol; components and concentration of primers and probes for separate PCRs for screening and determination. [file 1471-2334-14-27-S3.doc]

Table S3. PCR protocol; components and concentration of primers and probes for separate PCRs for screening and determination

|  | multiplex detection | identification PCRs | | |
| --- | --- | --- | --- | --- |
|  | OXA-48, VIM, IMP, |  |  |  |
| PCR | NDM, KPC, PhHV | OXA-48, CTX-M I-V, PhHV | VIM, IMP, PhHV | NDM, KPC, PhHV |
| components | µM | µM | µM | µM |
| CTX-M I-V F |  | 0.32* |  |  |
| CTX-M I-V R |  | 0.32* |  |  |
| CTX-M probe |  | 0.16 |  |  |
| OXA-48 F | 0.75 | 0.8 |  |  |
| OXA-48 R | 0.75 | 0.8 |  |  |
| OXA-48 probe | 0.1875 | 0.16 |  |  |
| VIM F | 0.75 |  | 0.6 |  |
| VIM R | 0.75 |  | 0.6 |  |
| VIM probe | 0.1875 |  | 0.14 |  |
| IMP F | 0.75 |  | 0.6 |  |
| IMP R1 | 0.375 |  | 0.3 |  |
| IMP R2 | 0.375 |  | 0.3 |  |
| IMP R3 | 0.75 |  | 0.6 |  |
| IMP probe1 | 0.2 |  | 0.16 |  |
| IMP probe2 | 0.2 |  | 0.16 |  |
| NDM F | 0.75 |  |  | 0.6 |
| NDM R | 0.75 |  |  | 0.6 |
| NDM probe | 0.25 |  |  | 0.2 |
| KPC F | 0.75 |  |  | 0.6 |
| KPC R | 0.75 |  |  | 0.6 |
| KPC probe | 0.25 |  |  | 0.2 |
| PhHV F | 0.375 | 0.2 | 0.2 | 0.2 |
| PhHV R | 0.375 | 0.2 | 0.2 | 0.2 |
| PhHV probe | 0.15 | 0.08 | 0.08 | 0.08 |
| Tris-HCl pH 8 | 375 | 375 | 375 | 375 |
| ^1^PCR reaction mix |  |  |  |  |
| PCR reaction volume | 20 µl | 25 µl | 25 µl | 25 µl |

PCR reaction mix^1^: (Sigma -Aldrich (E3004), Munich, Germany)

*concentration of each primer
